# Concurrent Diagnosis of Superficial Esophageal Cancer and Esophageal Achalasia: A Case Report and Literature Review

**DOI:** 10.1002/deo2.70164

**Published:** 2025-06-17

**Authors:** Ai Katsumi, Hideki Mori, Noriko Matsuura, Tatsuhiro Masaoka, Yusaku Takatori, Hideomi Tomida, Teppei Akimoto, Hiroko Ando, Motohiko Kato, Takanori Kanai

**Affiliations:** ^1^ Division of Gastroenterology and Hepatology Department of Internal Medicine Keio University School of Medicine Tokyo Japan; ^2^ Division of Research and Development for Minimally Invasive Treatment Cancer Center Keio University School of Medicine Tokyo Japan; ^3^ Center for Endoscopy Kawasaki Municipal Hospital Kanagawa Japan; ^4^ Center for Diagnostic and Therapeutic Endoscopy Keio University School of Medicine Tokyo Japan

**Keywords:** dysphagia | endoscopic submucosal dissection (ESD) | esophageal achalasia | esophageal cancer | peroral endoscopic myotomy (POEM)

## Abstract

We report a case of a 70‐year‐old woman with esophageal achalasia and concurrent superficial esophageal squamous cell carcinoma. Three adjacent superficial lesions were resected en bloc by endoscopic submucosal dissection (ESD), with no lymphovascular invasion. Given that the patient's dysphagia was effectively controlled with medication and dietary modifications, peroral endoscopic myotomy (POEM) was deferred following a careful assessment of the risk–benefit balance. As both ESD and POEM involve submucosal intervention, this case highlights the importance of individualized treatment based on symptom severity and lesion characteristics.

## 1 Introduction

Esophageal achalasia is an esophageal motility disorder characterized by failure of the lower esophageal sphincter (LES) and absence of normal esophageal peristalsis [1, 2]. Long‐standing achalasia increases the risk of esophageal squamous cell carcinoma [3]. The incidence of esophageal cancers was estimated at 0.078 and 0.28 per 100 person‐years from the onset and the diagnosis of the disease in the Japanese large database analysis, respectively [4]. Here, we present a case of superficial esophageal cancer diagnosed simultaneously with esophageal achalasia.

## 2 Case Report

A 70‐year‐old female was referred to our hospital with a 3‐year history of dysphagia. She was on a daily regimen of amlodipine 5 mg for essential hypertension and had no history of smoking or alcohol consumption.

**FIGURE 1:**
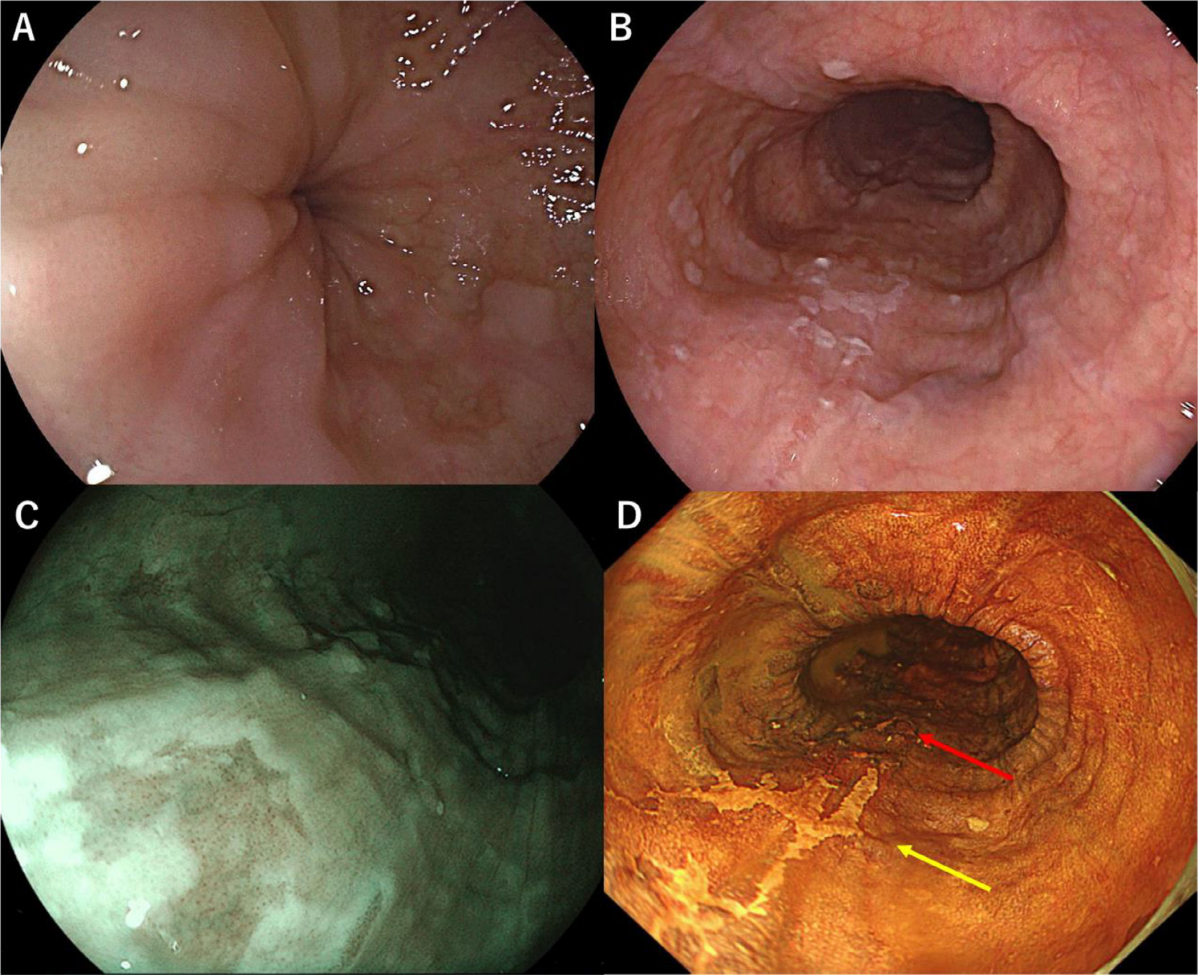
Endoscopic findings before endoscopic submucosal dissection. Esophagogastroduodenoscopy revealed characteristic findings of esophageal achalasia along with superficial esophageal cancer. (A) Esophageal Rosette at the esophagogastric junction (B) Two superficial, mesh‐like lesions on the posterior wall of the middle thoracic esophagus. (C) Under narrow‐band imaging, the lesion appears as a brownish area. (D) With iodine staining, a 24 × 20 mm 0‐IIc lesion (yellow arrow) and a 12 × 12 mm 0‐IIc lesion (red arrow) became clearly visible.

**FIGURE 2:**
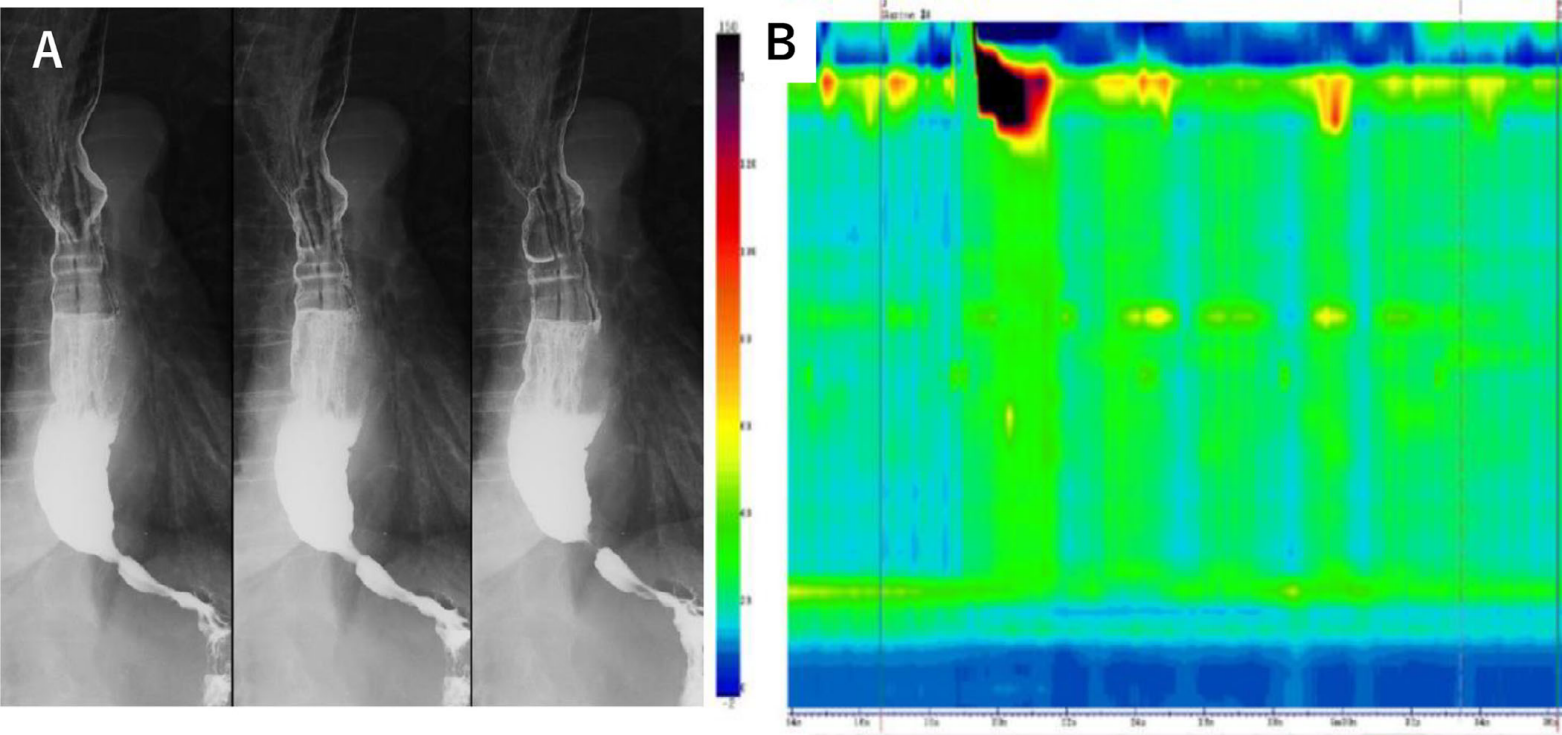
Physiological function tests for esophageal achalasia. Esophagography and esophageal manometry were performed to diagnose esophageal achalasia and identify its subtype. (A) Esophagography revealed esophageal dilation, retention of barium within the esophagus, and a smooth narrowing at the esophagogastric junction. (B) Esophageal manometry revealed failed relaxation of the lower esophageal sphincter, along with loss of peristalsis and pan‐esophageal pressurization.

Esophagogastroduodenoscopy revealed a characteristic “esophageal rosette” appearance in the lower esophagus (Figure 1A). Additionally, two slightly depressed lesions with a reticular pattern were observed on the posterior wall of the middle thoracic esophagus. The lesions measured 24 × 20 mm and 12 × 12 mm, located 30 and 32 cm from the incisors (Figure 1B). Under magnifying endoscopy using narrow‐band imaging (NBI), the tumor appeared as a brownish, irregular, depressed area (Figure 1C). Based on the Japanese Esophageal Society (JES) classification using magnifying NBI observation [5], the microvascular architecture of the lesion was classified as type B1, and the depth of invasion was diagnosed as cT1a‐EP/LPM. Although the lesion was identifiable despite vascular obscuration by keratinization of the esophageal epithelium, the delineation of its margins was not clearly defined. With iodine staining, the lesion exhibited a well‐demarcated border and revealed a reticular structure (Figure 1D). Histopathological examination of biopsy specimens confirmed a diagnosis of squamous cell carcinoma. Esophagography revealed barium retention in the esophagus, poor distension of the lower esophagus, and a maximum esophageal diameter of 3.6 cm (Figure 2A). Esophageal manometry using the Starlet system (Star Medical, Inc., Tokyo, Japan) demonstrated failed relaxation of the LES, with a median integrated relaxation pressure (IRP) of 24.1 mmHg, along with loss of peristalsis and panesophageal pressurization in 60% of swallows. The Eckardt score is a 0–12 grading system based on dysphagia, regurgitation, chest pain, and weight loss [6]. The patient's Eckardt score was 3, indicating daily dysphagia with occasional regurgitation, but no chest pain or weight loss. These findings led to a diagnosis of straight type, Type II achalasia based on the Chicago Classification (Version 4.0), considering that the median IRP was slightly below the threshold of 26.0 mmHg set by Kuribayashi et al., but remained borderline [1, 2]. The final diagnosis of achalasia was based on a comprehensive assessment, including esophagography findings (Figure 2B). Thus, the patient was diagnosed with simultaneous esophageal achalasia and superficial esophageal cancer.S1)

The patient's dysphagia was managed with diet and calcium channel blockers. As a result, peroral endoscopic myotomy (POEM) was deferred, and en bloc *r*esection of the superficial esophageal cancer by endoscopic submucosal dissection (ESD) was prioritized. Considering the limited lesion size, the risk of postoperative stricture was deemed low; however, careful attention was given to the potential worsening of esophageal motility and swallowing function due to preexisting achalasia.

**FIGURE 3:**
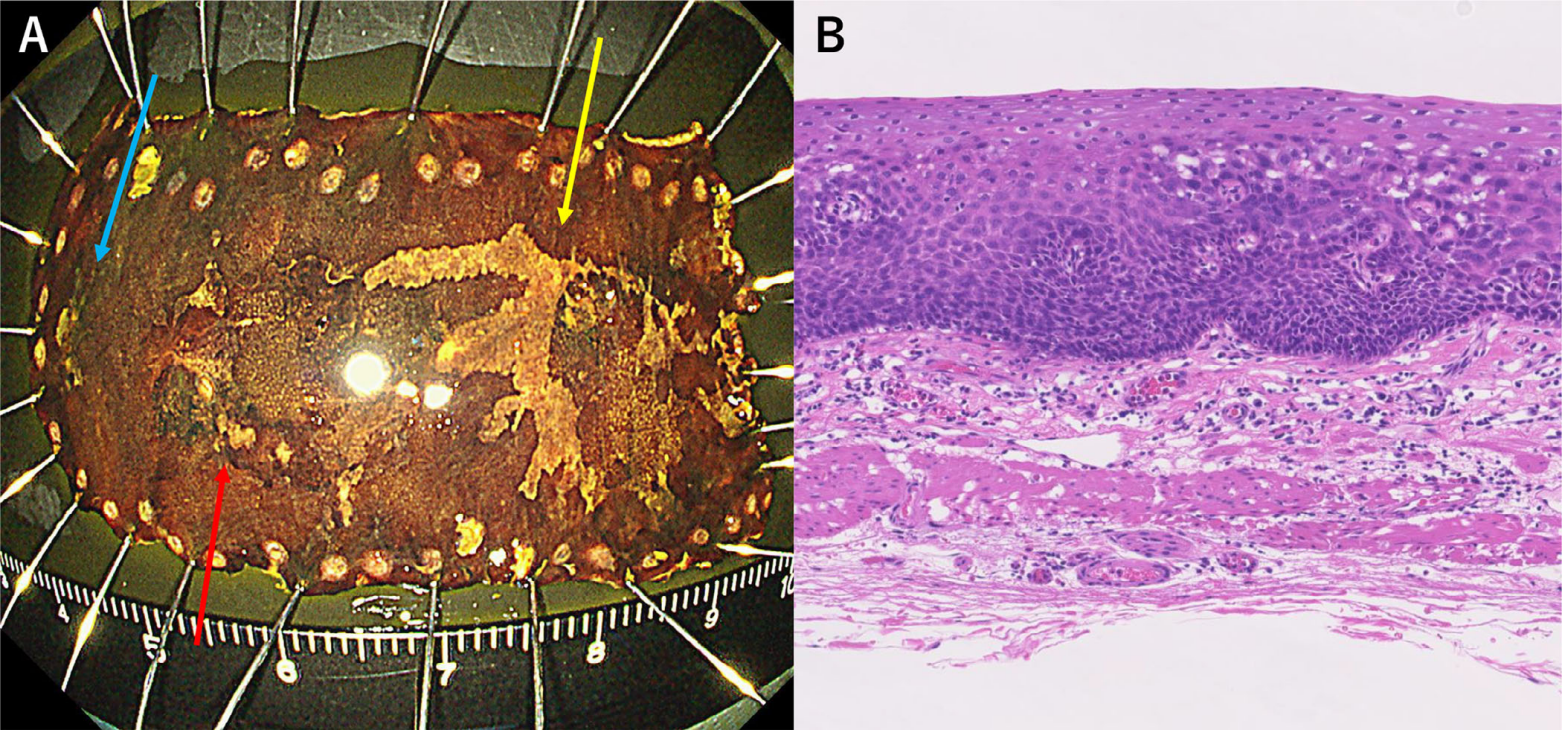
Pathological findings from endoscopic submucosal dissection (ESD) resection specimens. Pathological diagnosis was performed to assess the depth of invasion of the lesion resected by ESD and to confirm curative resection. (A) Three lesions were resected *en bloc* using ESD (yellow arrow, 0‐IIc, 24 × 20 mm; red arrow, 0‐IIc, 12 × 12 mm; blue arrow, 0‐IIc, 5 × 4 mm). (B) Hematoxylin and eosin staining, showing a mixture of hypodysplastic to intraepithelial carcinoma within the mucosa, with no evidence of invasion into the mucosal muscle plate, or vascular invasion.

ESD was performed without any complications. During the procedure, a third lesion measuring 5 × 4 mm was identified and also resected (Figure 3A). Histopathological examination confirmed all three lesions as squamous cell carcinoma in situ with no lymphovascular invasion (pT1a‐EP, ly0, v0; Figure 3B). Symptom monitoring during outpatient visits was conducted every 2 to 3 months for one year following ESD, during which no changes in dysphagia were observed, with Eckardt scores remaining at 3. A follow‐up endoscopy performed six months after ESD revealed no evidence of esophageal stricture, food retention, or other findings suggestive of worsening esophageal motility disorder. Additionally, no endoscopic signs of residual or recurrent esophageal cancer were detected. Endoscopic surveillance is scheduled at intervals of 6–12 months thereafter.

## 3 Discussion

This case highlights treatment planning for concurrent superficial esophageal cancer and achalasia. This is because ESD is the standard treatment for superficial esophageal cancer, while POEM is the standard treatment for esophageal achalasia [7]. Both procedures involve endoscopic access to the submucosal layer, necessitating careful consideration in determining the optimal sequence and approach to treatment. When early‐stage esophageal cancer and achalasia are diagnosed simultaneously, and both ESD and POEM are planned, there are three potential approaches: (1) performing ESD first, (2) performing POEM first, or (3) performing both procedures simultaneously.

Sato et al. reported a Japanese multicenter retrospective study of esophageal cancer cases treated before and after POEM, which compared ESD before and after POEM [8]. Endoscopic en bloc resection was performed in 95.8% and 89.3% of esophageal cancers diagnosed before and after POEM, respectively, with no statistically significant difference [8]; however, they caution that scarring after POEM can make ESD of esophageal cancer more challenging [8].

A literature search of PubMed and the Igaku‐Chuo‐Zasshi database from March 1983 to April 2025 using keywords “achalasia” AND “early esophageal cancer OR superficial esophageal cancer” identified 24 reported cases of esophageal achalasia combined with superficial esophageal cancer, including this case (Table 1). In five cases, achalasia treatment followed ESD/ endoscopic mucosal resection (EMR) using surgery or dilation; POEM was not used. The interval between ESD/EMR and achalasia treatment was relatively short, with a median of two months. In most cases, a two‐stage therapeutic strategy appeared to have been planned from the outset. In 19 cases, treatment for achalasia preceded or was conducted simultaneously with endoscopic treatment for cancer. In five, POEM and EMR/ESD were simultaneous; in two, the POEM incision required adjustment due to overlap.

**TABLE 1:** Case reports of esophageal achalasia and superficial esophageal cancer.

Author	Year	Age/ sex	History of achalasia (year)	Achalasia type	Achalasia treatment	EC location	Gross subtype of EC	Size of EC	Depth of EC	EC treatment	Timing of the treatment of achalasia
Eckardt^a^	1992	42F	N/A	N/A	Surgery	Upper	N/A	N/A	N/A	N/A	14 years before surgery
Watanabe^b^	1996	70F	13	Sigmoid	EBD	Upper	0‐IIc	N/A	LPM	EMR	13 years before EMR
Chino^c^	1997	70F	13	Sigmoid	N/A	Upper	0‐IIc	N/A	MM	EMR	N/A
Saeki^d^	2000	49M	30	Sigmoid	EBD	Upper	0‐IIc	60	LPM	EMR	2 weeks before EMR
Koyama^e^	2000	73M	0.5	Spindle	EBD	Middle/Lower	0‐IIc / 0‐IIc	15/15	EP/EP	EMR	3 weeks before EMR
Hamamoto^f^	2004	68M	40	Sigmoid	EBD	Lower	0‐IIc	10	LPM	EMR	3 weeks after EMR
Chino^g^	2008	65M	13	Sigmoid	Surgery	Upper	0‐IIb	10	EP	EMR	2 years after ESD
Akimoto^h^	2011	60M	33	Sigmoid	Surgery	Upper	0‐IIc	10	LPM	ESD	3 years before ESD
Oota^i^	2012	63F	N/A	N/A	Surgery	Middle	0‐IIc	20	MM	ESD	40 years before ESD
Yamamoto^j^	2012	67M	20	Sigmoid	Surgery	Middle	0‐IIc	10	LPM	ESD	N/A
Mori^k^	2013	81M	36	Sigmoid	Surgery	Lower/Upper/Middle	0‐IIc/0‐IIa+0‐IIc+0‐IIb/ 0‐IIc	30/70/10	EP/SM2‐3/EP	APC/CRT/ESD	17 years before APC
Tsuboi^l^	2013	63M	15	Flask	Surgery	Middle/Middle	0‐IIc/ 0‐IIa	20/10	N/A	ESD	2 months after ESD
Chino^m^	2014	46M	9	Flask	Surgery	Middle/Middle	0‐IIb/ 0‐IIc	10/10	N/A	ESD	1 year before ESD
Okazaki^n^	2014	70F	22	N/A	EBD	Middle	0‐IIc	50	EP‐LPM	ESD	Before ESD
Tang^o^	2015	41F	1	N/A	POEM	Middle	N/A	N/A	EP‐LPM	EMR	At the same time
Tabuchi^p^	2016	74F	20	Sigmoid	Surgery	Upper Middle	0‐IIc	50	LPM	ESD	4 months after ESD
Shi^q^	2017	50M	6	Sigmoid	POEM	Upper 1/Lower 2	N/A	15/10/15	EP‐LPM	ESD	At the same time
Maruyama^r^	2018	55M	18	Type I	EBD	Upper 1/Middle 5	All 0‐IIc	19/20/20/15/9/54	MM/EP/EP/EP/LPM/LPM	ESD	3 weeks after ESD
Lee^s^	2023	72M	5	Type I	EBD, POEM	Middle	0‐IIc	15	LPM	ESD	At the same time
Kamio^t^	2024	75F	N/A	Type II	POEM, ESD	Upper	0‐IIc	10	EP	ESD	3 months after POEM
Zhao^u^	2024	62F	10	Sigmoid	POEM, ESD	Middle	0‐IIc	N/A	LPM	ESD	At the same time
Li^v^	2024	32M	1	N/A	POEM, Surgery	Middle	0‐IIc	N/A	EP	Surgery	11 months after POEM
Chen^w^	2024	65M	20	N/A	POEM, ESD	Lower	0‐IIc	18	EP	ESD	At the same time
Present case	2024	70F	3	Straight, Type II	N/A	Middle	All 0‐IIc	5/20/12	EP‐LPM	ESD	N/A

Abbreviations: CRT, chemoradiotherapy; EBD, endoscopic balloon dilation; EC, esophageal cancer; EMR, endoscopic mucosal resection; EP, epithelium; ESD, endoscopic submucosal dissection; LPM, lamina propria mucosae; MM, muscularis mucosae; PC, argon plasma coagulation; POEM, peroral endoscopic myotomy.

The classification of esophageal achalasia in references b, c, d, e, f, g, h, j, k, l, m, p, q, and u was based on esophagography findings, while that in references r, s, and t was based on high‐resolution esophageal manometry findings.

The classification of achalasia could not be confirmed in the cases reported in references a, i, v, and w.

In patients diagnosed with both esophageal achalasia and superficial esophageal cancer, treatment strategy should be individualized based on the severity of achalasia symptoms and endoscopic visibility. In cases with marked dysphagia, performing POEM prior to ESD can alleviate symptoms, reduce the risk of aspiration, and improve endoscopic visualization by minimizing food stasis. This, in turn, may enhance the diagnostic accuracy for esophageal cancer. Conversely, in patients with mild achalasia symptoms and a clear endoscopic view, prioritizing ESD may be more appropriate to facilitate early cancer treatment.

Simultaneous POEM and ESD may be considered to minimize the overall treatment burden; however, this approach requires careful planning due to potential procedural risks. When the POEM incision site and the ESD resection area are in close proximity, there is an increased risk of complications such as esophageal stricture and perforation. This risk may be particularly pronounced when both are performed at the same esophageal level, although supporting data remain limited.

The feasibility of performing both POEM and ESD also depends on the spatial relationship between the POEM tunnel and the tumor site [8]. If the POEM incision line does not overlap with the lesion targeted for resection, both procedures can generally be performed without difficulty. In contrast, when overlap is anticipated, modifying the POEM approach—for instance, by adjusting the incision site—may allow for the safe execution of both treatments. In cases with substantial overlap, ESD over the POEM tunnel remains technically feasible but challenging, and should only be attempted following a thorough assessment of the associated risks and resectability.

In this case of coexisting esophageal achalasia and superficial esophageal cancer, the patient's dysphagia was mild (Eckardt score of 3), non‐progressive, and adequately managed with dietary modification and calcium channel blockers. This clinical course allowed prioritization of cancer treatment. The cancer lesion extended from the left to the posterior wall of the mid‐thoracic esophagus, involving approximately one‐quarter of the circumference. Given the diagnosis of type II achalasia [9], we anticipated that the required myotomy length for POEM would be minimal. Although ESD‐related fibrosis was expected, its impact on a future POEM via the anterior wall approach was considered negligible. Thus, POEM was deferred, with plans to reconsider it if dysphagia progressed [10].

While POEM can provide short‐term symptom relief, its indication must be balanced against risks such as perforation, bleeding, and reflux esophagitis [8]. Furthermore, from a long‐term perspective, POEM may improve esophageal clearance and potentially reduce the risk of metachronous cancer development in achalasia patients, which is believed to arise from chronic mucosal inflammation [3]. However, current evidence remains inconclusive as to whether achalasia interventions, including POEM and fundoplication, reduce the incidence of esophageal cancer.

In conclusion, treatment of patients with both esophageal achalasia and esophageal cancer requires an individualized approach based on achalasia severity and esophageal cancer extent and location.

## Conflicts of Interest

The authors declare no conflicts of interest.

## Ethics Statement

All procedures were performed in accordance with the ethical standards of the Declaration of Helsinki and its later amendments.

## Consent

Informed consent was obtained from the patient for the publication of this case report.

## Supporting information



Supplementary Material: References for Case Reports
