# Supplementary material for: Concurrent Diagnosis of Superficial Esophageal Cancer and Esophageal Achalasia: A Case Report and Literature Review
Source: DEN Open. 2025 Jun 17;6(1):e70164. doi: 10.1002/deo2.70164 (PMC12171625; doi:10.1002/deo2.70164)
Supplement: Supplementary file 1 — Supplementary Material: References for Case Reports [file DEO2-6-e70164-s001.docx]

Supplementary Material

References for Case Reports

a. Eckardt VF, Aignherr C, Bernhard G. Predictors of outcome in patients with achalasia treated by pneumatic dilation. *Gastroenterology.* 1992;103:1732-1738.

b. Watanabe J, Wada T, Deguchi R, et al. Superficial carcinoma of the esophagus complicated with achalasia cured by endoscopic mucosal resection. *Gastroenterol Endosc　(Jpn).* 1996;38:2415-2419.

c. Chino O, Makuuchi H, Kanno K, et al. Endoscopic diagnosis of esophageal carcinoma complicated with achalasia. *Gastroenterol Endosc (Jpn).* 1997;39:1052-1061.

d. Saeki T, Tanabe S, Ishii K, et al. Extensive type 0-IIc esophageal carcinoma with achalasia. *Gastroenterology (Jpn).* 2000;35:1293-1297.

e. Koyama T, Miyata Y, Tomori A, et al. Multiple early-stage carcinoma of the esophagus associated with achalasia. *Gastroenterology (Jpn).* 2000;35:1281-1285.

f. Hamamoto T, Maeda S, Noguchi M, et al. Vigorous achalasia associated with superficial carcinoma of the esophagus. *Jpn J Gastroenterol (Jpn).* 2004;101:983-988.

g. Chino O, Shimada H, Kise Y, et al. Early carcinoma of the esophagus with achalasia treated by endoscopic mucosal resection. *Tokai J Exp Clin Med (Jpn).* 2008;33:13-16.

h. Akimoto T, Komura N, Yajima H, et al. Early esophageal cancer after surgical treatment for achalasia. *J Jpn Surg Assoc (Jpn).* 2011;72:2261-2265.

i. Oota M, Narimiya K, Kudo K, et al. Early esophageal cancer occurring 40 years after surgery for achalasia. *Surgery (Jpn).* 2012;66:1917-1919.

j. Yamamoto S, Kobayashi N, Yano F, et al. Superficial carcinoma of the esophagus arising in achalasia. *Jpn J Coll Surg (Jpn).* 2012;37:946-950.

k. Mori K, Koyanagi K, Hiraiwa N, et al. Multiple superficial carcinoma of the esophagus after achalasia. *Prog Dig Endosc (Jpn).* 2013;82:102-103.

l. Tsuboi K, Omura N, Yano F, et al. Laparoscopic surgery after endoscopic submucosal dissection in achalasia with early carcinoma: a case report. *Jikeikai Med J (Jpn).* 2013;60:35-38.

m. Chino O, Makuuchi H, Ozawa S, et al. Multiple early carcinoma of the esophagus with achalasia treated by endoscopic submucosal dissection. *Ann Cancer Res Ther.* 2014;22:1-5.

n. Okazaki J, Kitamura S, Nakamura F, et al. Early esophageal cancer complicated by achalasia. *Clin Dig Tract (Jpn).* 2015;20:71-74.

o. Tang X, Ren Y, Jiang B, Gong W. Endoscopic mucosal resection for esophageal dysplasia in an achalasia patient followed by peroral endoscopic myotomy. *J Gastroenterol Hepatol.* 2015;30:1563.

p. Tabuchi S, Koyanagi K, Nishimura M, et al. Endoscopic submucosal dissection of superficial esophageal cancer associated with achalasia. *Gastroenterol Endosc (Jpn).* 2017;59:1403-1408.

q. Shi S, Fu K, Dong X-Q, Hao Y-J, Li S-L. Concurrent endoscopic submucosal dissection and modified peroral endoscopic myotomy for achalasia with early neoplasms. *World J Gastrointest Endosc.* 2017;9:99-104.

r. Maruyama K, Tominaga K, Sakai D, et al. Multiple superficial esophageal cancers with achalasia treated by endoscopic submucosal dissection. *Gastroenterol Endosc (Jpn).* 2018;60:1076-1082.

s. Lee M-H, Jung K, Kim J-H, et al. Concomitant endoscopic treatment of achalasia with superficial cancer. *Korean J Gastroenterol.* 2023;82:248-253.

t. Kamio T, Hirata S, Hamada K, et al. A Case of Esophageal Squamous Cell Carcinoma

Detected After Peroral Endoscopic Myotomy in a Patient With Achalasia. *Cureus.* 2024;16:e71604.

u. Zhao C, Li L, Chai N. Peroral endoscopic myotomy for sigmoid-type achalasia after Heller myotomy and endoscopic submucosal dissection for an early esophageal cancer in a single endoscopic procedure. *Endoscopy.* 2024;56(S 01):E839-E840.

v. Li S, Chen X, Zhang L, et al. Early-onset esophageal squamous cell carcinoma with achalasia: A case report. *Medicine (Baltimore).* 2024;103:e37140.

w. Chen ZH, Guo LL, Wang LS, et al. A case of achalasia associated with early esophageal cancer. *Rev Esp Enferm Dig.* 2024;116:653-654.
